# Nonseminomatous germ cell tumor with seizure disorder and mental retardation

**DOI:** 10.4103/0970-2113.56351

**Published:** 2009

**Authors:** Vinaya Karkhanis, J. M. Joshi

**Affiliations:** *Department of Respiratory Medicine, T. N. Medical College, BYL Nair Hospital, Mumbai, India*

**Keywords:** Klinefelter syndrome, nonseminomatous germ cell tumor, Mediastinal germ cell tumors

## Abstract

We report a case of a patient who presented with anterior mediastinal mass, seizure disorder, and mental retardation. Computerized tomography–guided fine-needle aspiration biopsy of the mass showed nonseminomatous germ cell tumor. Chromosomal analysis showed XXY karyotype. A diagnosis of Klinefelter syndrome and mediastinal germ cell tumor was made.

## INTRODUCTION

A diagnosis of Klinefelter syndrome is rarely made before puberty due to the subtle manifestations in childhood and under-recognition by health care practitioners.[1] Learning disabilities and school-adjustment problems are often viewed as “part of growing up” or attention deficit disorder.[2] Recognition and identification of early clinical characteristics are very important.[3] Primary anterior mediastinal neoplasms comprise a diverse group of tumors and account for 50% of all mediastinal masses. A variety of mediastinal germ cell tumors — teratomas, seminomas, nonseminomatous germ cell tumors — account for 10% to 15% of mediastinal tumors in adults; and 25%, in children.[4] We present a case of Klinefelter syndrome and mediastinal germ cell tumor and discuss the association between the two.

## CASE REPORT

A 14-year-old boy was referred with history of dry cough, dyspnea, and fever of three months' duration, with seizure disorder since the age of two years. He also had delayed developmental milestones and poor scholastic performance. Physical examination revealed height and weight of 147 cm and 47 kg, respectively, which were below 5 percentile for his age. There was presence of gynacomastia. Vital parameters were within normal limits. Respiratory examination showed bulging of the chest wall with decreased breath sounds all over the right hemithorax. On further examination, a testicular swelling was noted.

**Figure 1:**
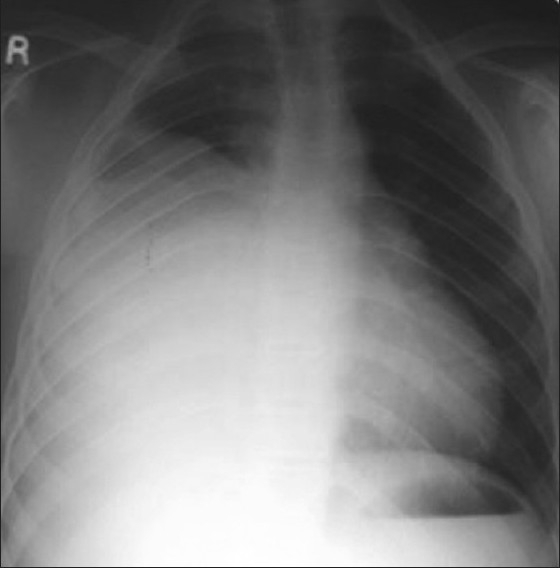
X-ray chest showing anterior mediastinal mass

**Figure 2:**
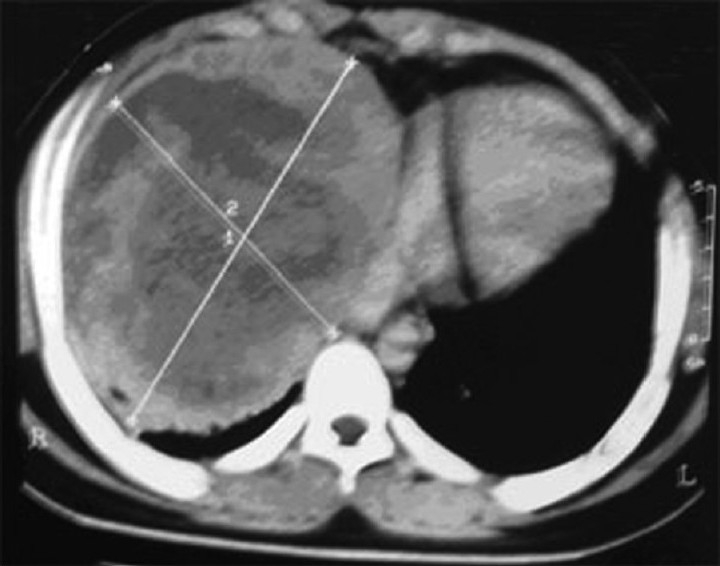
Computed tomography thorax showing inhomogeneously enhancing mass lesion involving the entire right hemithorax

**Figure 3:**
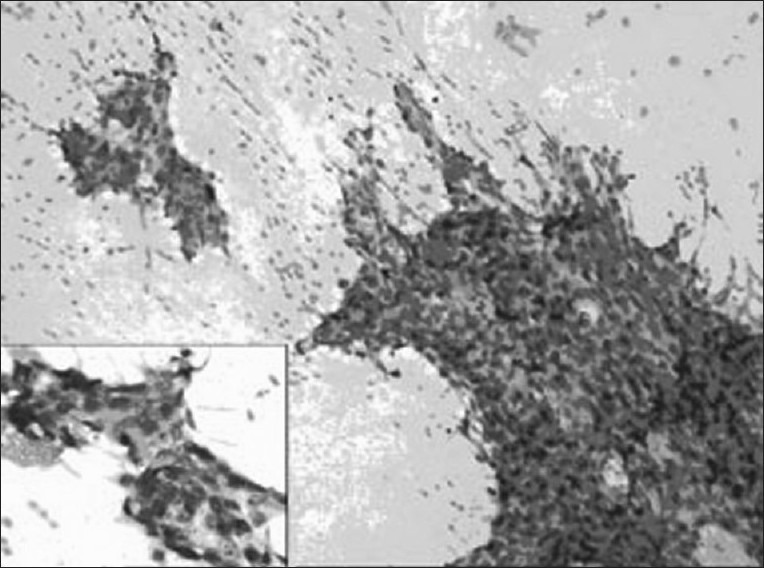
Photomicrograph (H and E, ×10 and ×20) of computed tomography-guided fine-needle aspiration cytology of the tumor showing cohesive clusters of tumor cells. Inset reveals vacuolated cytoplasm of tumor cells

**Figure 4:**
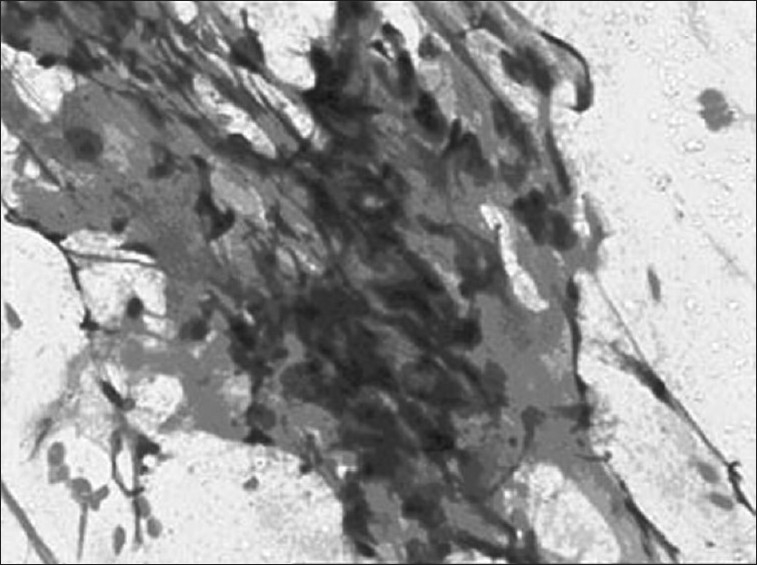
Photomicrograph (H and E, ×20) showing hyaline basement membrane–like material within cell clusters

Investigations showed hemoglobin - 10.6 g/dL, white blood count - 12600/μL, blood urea nitrogen- 10 mg%, Lactate dehydrogenase (LDH) – 827IU/L; human immunodeficiency virus (HIV) was nonreactive. Chest X-ray showed anterosuperior mediastinal mass [Figure 1]. Ultrasonography (USG) showed large hypo-echoic mass with anechoic areas within it. On computed tomography (CT) of thorax, there was inhomogeneously enhancing mass lesion 31-36 HU and 95 HU on contrast study [Figure 2], almost involving the entire right lung, with relative sparing of the apex, extending into the prevascular space and partially compressing the right main bronchus. Ultrasonography of scrotum showed left testis measuring 2.5 × 1.7 × 0.7 cm with 6 × 6 mm well-defined hypo-echoic lesion with specks of calcification in the wall. Right testis measured 2.2 × 1.6 × 0.9 cm with few specks of calcification. Fine-needle aspiration biopsy of the mediastinal mass showed malignant cells with large nuclei and high nucleo-cytoplasmic ratio and hyperchromatic nuclei, suggestive of nonseminomatous germ cell tumor [Figures 3 and 4]. Human chorionic gonadotropin (HCG) hormone and alfa fetoproteins (AFPs) were 1.65 mIU/ mL and 6553.94 ng/mL, respectively. Intelligence quotient analysis score was 70, suggestive of mild mental retardation. He was subsequently investigated for history of seizure disorder, delayed developmental milestones, poor scholastic performance, mental retardation, and gynacomastia. Electroencephalogram showed normal wave pattern. Chromosomal analysis done using GTG-banding technique using Giemsa showed cytogenic profile of 3 sex chromosomes and 44 autosomes, with karyotype 47 XXY in all metaphases studied, suggestive of Klinefelter syndrome. The patient expired after five cycles of chemotherapy with bleomycin, cisplatin, etoposide.

## DISCUSSION

**Figure 5:**
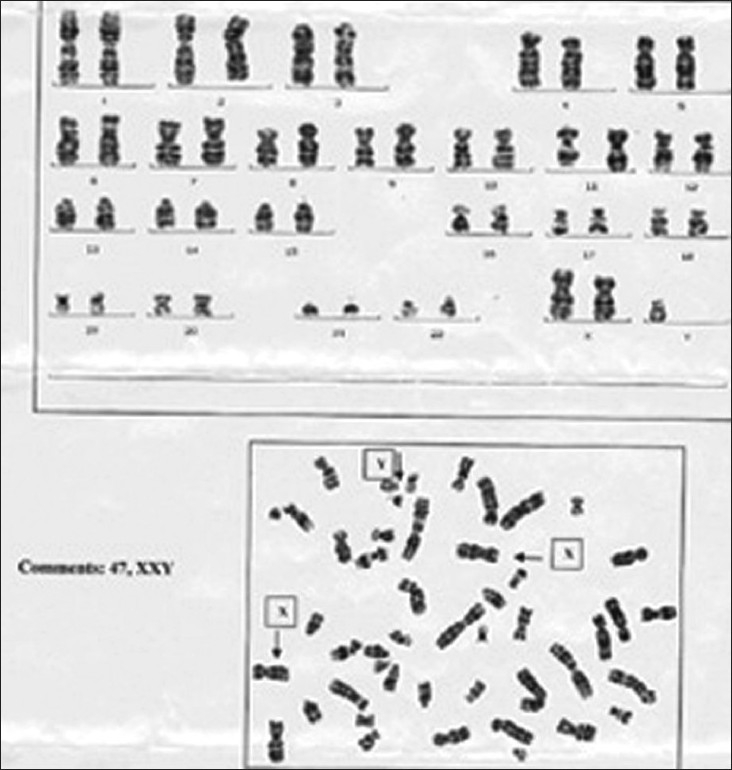
Chromosomal analysis

Mediastinal germ cell tumors are a heterogeneous group of benign and malignant neoplasms thought to originate from primitive germ cells “misplaced” in the mediastinum during early embryogenesis. The anterior mediastinum, especially the anterosuperior position, is the most common extragonadal primary site. Lactate dehydrogenase and serological markers such as AFPs and beta HCG are frequently positive.[5] In a series of patients with extragonadal germ cell tumors, 71% patients with nonseminomatous germ cell tumors had elevated AFP levels, and 54% had elevated beta HCG levels. Radiologically; these are large, irregular, anterior mediastinal masses, often with extensive, central, irregular, and heterogenous areas of low attenuation due to necrosis, hemorrhage, and cyst formation. Pleural and pericardial effusions are common.[6] CT scan frequently shows an inhomogeneous mass with multiple areas of hemorrhage and necrosis, differing from the usually homogeneous appearance of mediastinal seminoma. Our patient presented with inhomogeneous anterior mediastinal mass with multiple areas of hemorrhage and necrosis on CT scan examination, which was diagnosed to be a nonseminomatous germ cell tumor on histopathology. AFP and beta HCG levels were elevated. Subsequent chromosomal analysis showed cytogenic profile of 3 sex chromosomes and 44 autosomes with karyotype 47 XXY in all the metaphases studied, suggestive of Klinefelter syndrome [Figure 5].

In a recent study, DNA samples from 1205 patients of mental retardation of unknown etiology were screened for fragile X syndrome, and all were detected to have Klinefelter syndrome, emphasizing the importance of cytogenic testing of all prepubertal males with cognitive impairment, even without dysmorphic features.[7] In most cases, the physical and neurobehavioral characteristics of Klinefelter syndrome are relatively mild and are not usually associated with moderate-to-severe mental retardation.[8] Whereas a higher tumor risk in this condition is controversial, Klinefelter syndrome has a relative risk of 66.7% for malignant mediastinal germ cell tumor. Approximately 20% of nonseminomatous malignant mediastinal germ cell tumors are associated with Klinefelter syndrome — 50 times the expected frequency.[9] Average age of patients with Klinefelter syndrome who develop extragonadal germ cell tumors is 18 years. Mediastinal tumors that produce bioactive substances may be associated with gynacomastia and precocious puberty. Klinefelter syndrome has been rarely associated with testicular microlithiasis and has a predisposition to testicular germ cell neoplasm.[10]

Bukowski and associates of the southwest oncology group treated 41 patients with extragonadal seminoma or nonseminomatous malignant germ cell tumors with four cycles of chemotherapy containing bleomycin, cisplatin, and various other agents, followed by surgical resection. The two- and five-year survival rates for the patients in this series were 67% and 60%, respectively.[11] Currently the standard treatment for primary mediastinal malignant germ cell tumors is four cycles of chemotherapy with bleomycin, etoposide, and cisplatin. Higher cure rates are expected in cases of metastatic nonseminomatous germ cell tumors. More conclusive data would require a larger randomized trial.[12]

Recognition, identification of early clinical characteristics and evaluation of mediastinal germ cell tumor for Klinefelter syndrome are important. Chest physicians should be aware of this association since they are often the first to evaluate patients with mediastinal masses.
